# Modulation of Immune Tolerance via Siglec-Sialic Acid Interactions

**DOI:** 10.3389/fimmu.2018.02807

**Published:** 2018-12-07

**Authors:** Joyce Lübbers, Ernesto Rodríguez, Yvette van Kooyk

**Affiliations:** Molecular Cell Biology and Immunology, Amsterdam UMC, Vrije Universiteit Amsterdam, Cancer Center Amsterdam, Amsterdam Infection and Immunity Institute, Amsterdam, Netherlands

**Keywords:** mononuclear phagocytes, dendritic cells, macrophages, Siglecs, tolerance, inflammation, sialic acid, cancer

## Abstract

One of the key features of the immune system is its extraordinary capacity to discriminate between self and non-self and to respond accordingly. Several molecular interactions allow the induction of acquired immune responses when a foreign antigen is recognized, while others regulate the resolution of inflammation, or the induction of tolerance to self-antigens. Post-translational signatures, such as glycans that are part of proteins (glycoproteins) and lipids (glycolipids) of host cells or pathogens, are increasingly appreciated as key molecules in regulating immunity vs. tolerance. Glycans are sensed by glycan binding receptors expressed on immune cells, such as C-type lectin receptors (CLRs) and Sialic acid binding immunoglobulin type lectins (Siglecs), that respond to specific glycan signatures by triggering tolerogenic or immunogenic signaling pathways. Glycan signatures present on healthy tissue, inflamed and malignant tissue or pathogens provide signals for “self” or “*non-self”* recognition. In this review we will focus on sialic acids that serve as “self” molecular pattern ligands for Siglecs. We will emphasize on the function of Siglec-expressing mononuclear phagocytes as sensors for sialic acids in tissue homeostasis and describe how the sialic acid-Siglec axis is exploited by tumors and pathogens for the induction of immune tolerance. Furthermore, we highlight how the sialic acid-Siglec axis can be utilized for clinical applications to induce or inhibit immune tolerance.

## Highlights

- Siglecs have an immune modulatory effect on TLR signaling.
- Sialic acids can be presented by pathogens through synthesis of “mimic” structures or the novo synthesis for survival advantage.
- Hyper sialic acid expression in the tumor microenvironments is linked to immune suppression.
- Targeting the sialic acid-Siglec axis could have beneficial effects in therapy in cancer, allergies and auto immune diseases.

## Introduction

The human mononuclear phagocyte network consists of monocytes, different subsets of macrophages (MQ) and Dendritic cells (DCs) depending on their origin and tissue micro-environment. In each microenvironment, differentiation is dictated by various components such as stromal cell compartment, presence of immune cells and the diversity of chemokines and cytokines present (1). Moreover, mononuclear phagocytes are key instructors for inflammatory or tolerogenic programming of the immune system. The presence of MQ and DCs at multiple sites in the human body, like gut, lung, brain, oral mucosa, lymphnodes, spleen, skin and peripheral blood illustrates their importance in controlling immunity and tolerance (2–5). MQ are plastic cells that can polarize according to the signals they receive and this polarization is mainly described as classical activated M1, alternatively activated M2, or tumor-associated macrophages (TAM). The M1, depicted as pro-inflammatory cells, are induced by stimulating MQ with LPS and/or IFN-γ that produce IL-1, TNF-α and nitric oxide. On the other hand, M2 MQ are induced by stimulation with IL-4 and have anti-inflammatory and tissue repair properties, producing Il-10 and TGF-ß (6, 7). TAMs are found in the microenvironment of tumors, promoting tumor growth by among others release angiogenic factors like VEGF and EGF, attract regulatory T cells and inhibit effector T cells by the release of multiple cytokines and chemokines such as IL-10, TGF-ß, and CCL22 (8). DCs consists also of multiple subsets, were the conventional DCs (cDCs) and plasmacytoid DCs (pDCs) are the main populations in peripheral blood. The cDCs are the main antigen-presenting subset, able to present antigens to and activate antigen specific naïve CD4^+^ and CD8^+^ T cells and are able to secrete multiple proinflammatory and anti-inflammatory cytokines like IL12p70 and IL-10, respectively (9, 10). pDCs do not prime naïve T cells, however, there specialized function is the production of type I interferon (IFN-α/β) in response to viruses (11). Different cytokines like IFN-α, TNF-α and LPS can polarize cDCs into a more immunogenic state (12, 13), while other cytokines like IL-10 and TGF-ß induce tolerogenic cDCs that express checkpoint ligands like PD-L1 and produce the checkpoint molecule indoleamine 2,3-dioxygenase (IDO). *In-vitro* treatment of DCs with dexamethasone or vitamin D3 will also result in tolerogenic DCs (14). Functionally the main characteristics of MQ is their phagocytic capacity, while DCs are key in antigen presentation and stimulation of naïve T cells into antigen-specific effector T cells, however, some of these functions are not 100% restricted and are also shared between MQ and DCs.

*In-vitro*, human monocyte-derived DCs (moDCs) and monocyte-derived MQ (moMQ) can be generated from monocytes. Culturing monocytes with GM-CSF and IL-4 gives rise to moDCs, while culturing monocytes with M-CSF or GM-CSF alone creates moMQ (15, 16). moDCs and moMQ are often used as model systems for inflammatory DCs and MQ, respectively, as they are easily obtained in large numbers. moDCs are excellent antigen presenters, and able to induce antigen-specific CD4^+^ and CD8^+^ T cells, while culturing with IL-10 or TGF-β generates moDC prone to induce tolerance (4, 17–21). However, recent studies using mass cytometry as well as single cell RNA sequencing have revealed that moDCs are distinct from human peripheral blood and skin-derived DCs (2, 22).

Mononuclear phagocytes have an important function in maintenance of tissue homeostasis and the resolution of inflammation. They express multiple pattern recognition receptors (PRRs), like toll like receptors (TLR) and CLRs to recognize pathogen-associated molecular patterns (PAMPs), damaged self-antigens (DAMPs) or altered glycosylated self-antigens, such as tumor antigens (3, 23). The differentiation and maturation status of mononuclear phagocytes alters the expression levels of PRR (24, 25). CLRs is a large family of glycan-specific receptors that include, amongst others: DC-SIGN (CD209), Mannose receptor (MR, CD206), DEC-205 (CD205), Dectin-1, Macrophage galactose-type lectin (MGL, CD301) and Langerin (CD207) (26, 27). These CLRs are glycan-binding receptors, recognizing a wide variety of carbohydrate structures, like fucoses and mannoses found on host glycoproteins expressed by cells or pathogens or β-glucan structures that are only expressed on pathogens such as *Aspergillus fumigatus* and *Saccharomyces cerevisiae* (27–29). CLRs play an important role in the antigen uptake for processing and presentation of peptides on MHC class I and II, thereby stimulating antigen-specific T cell responses and T helper differentiation (27). Some CLRs, like Dectin-1, have the ability to directly modulate the DC or MQ phenotype and cytokine responses, while, other CLRs, like DC-SIGN and MGL are also highly expressed on tolerogenic DC/MQ and modulate TLR signaling through the acetylation of p65 and the induction of IL10 production (30–32).

**Figure 1:**
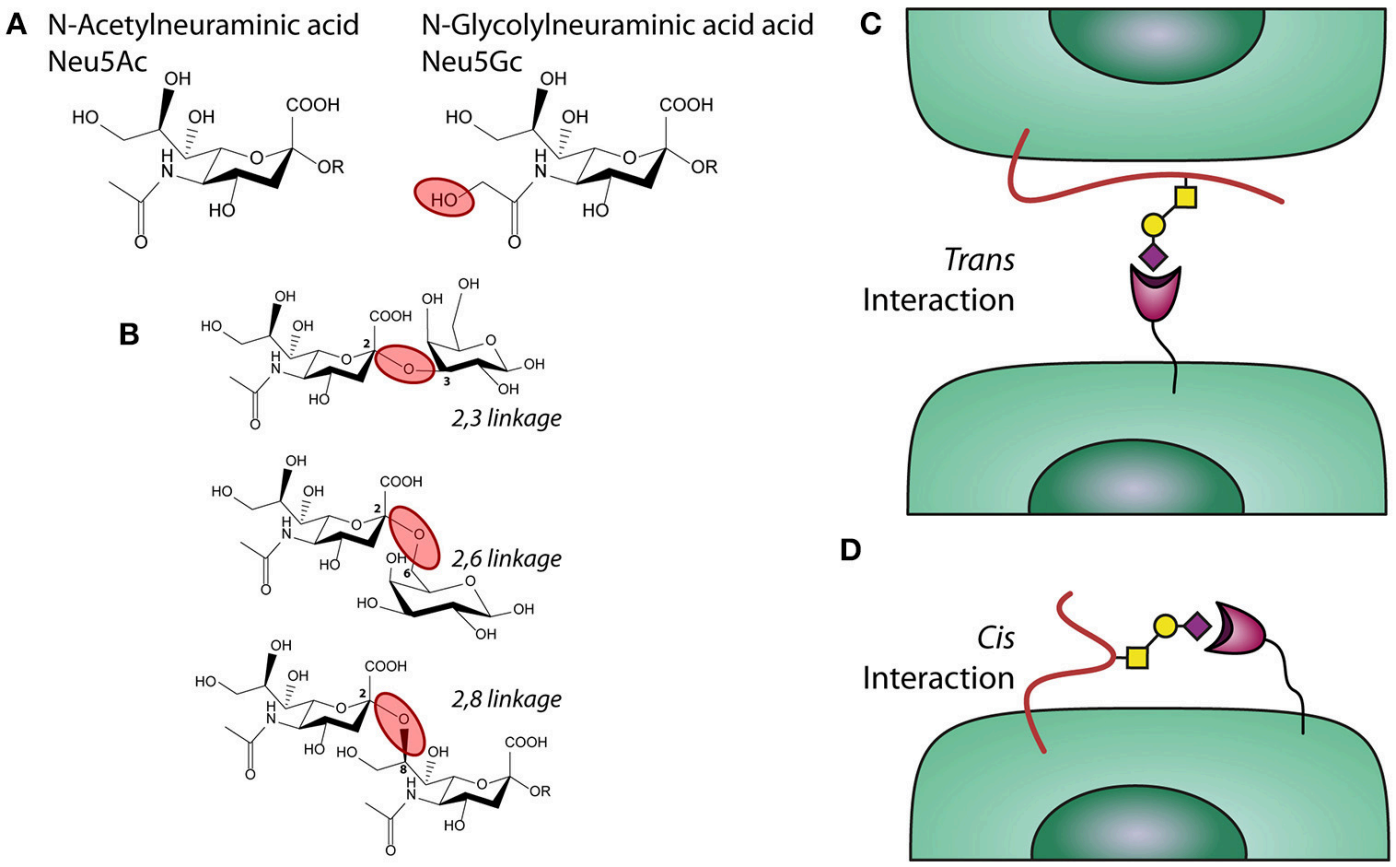
Sialic acids, linkages, and interactions. **(A)** chemical structure of sialic acids Neu5Ac and Neu5Gc. **(B)** α2,3; α2,6, and α2,8 linked sialic acids. **(C)** Trans and **(D)** Cis interactions of Siglecs with sialic acids.

Next to TLRs and CLRs, mononuclear phagocytes express Sialic acid binding immunoglobulin type lectins (Siglecs), that recognize sialic acids, a family of sugars with a nine-carbon sugar core structure derived from neuraminic acid, with the N-acetylneuraminic acid (Neu5Ac) being the main moiety present in humans (Box 1 and Figure 1). Sialic acids are generally the last sugars added during the glycosylation process, thereby capping a diverse array of glycosylation structures (44, 45). Often, the presence of sialic acids functions as a self-associated molecular pattern (SAMP) and thus, Siglecs can serve as sensors for “self” (46). Most Siglecs possess an intracellular immunoreceptor tyrosine-based inhibition motif (ITIM) that induce strong inhibitory signaling when Siglecs bind sialic acids (47). Interestingly, both pathogens and tumor cells use enhanced expression of sialic acids as a mechanism to modify the immune system in their favor, illustrating that the sialic acid-Siglec axis is a key regulator in infection and cancer.

Box 1 **Sialic acid.**

Sialic acids are a family of sugars with nine carbons derived from neuraminic acid that are negatively charged. Humans are able to synthetize Neu5Ac (Figure 1A), while other mammals can also synthetize the structure N-glycolylneuraminic acid (Neu5Gc). A deletion in the gene encoding the enzyme CMAH (Cytidine monophosphate-N-acetylneuraminic acid hydroxylase) is the reason why humans cannot produce Neu5Gc (33).

**Synthesis**

The expression of sialylated glycans is the result of glycosylation related enzymes able to catalyse the addition or removal of a glycan to growing carbohydrate structures. The transfer of sialic acid motifs from an activated donor (CMP-NeuAc, **C**ytidine 5′-**M**ono**P**hospho-N-**Ac**etyl**N**euraminic acid) to underlying glycans that serve as acceptors, is performed by a group of enzymes called sialyltransferases. Humans express more than 20 different sialyltransferases, each differing in their tissue expression, substrate specificity and linkages produced (34). The synthesis of sialylated structures depends also on the presence of the donor, which is synthetized in the nucleus by the enzyme CMAS (CMP-Neu5Ac synthetase) and subsequently transported into the Golgi via the transporter SLC35A1 (33, 35). Sialic acid blocking glycomimetic: Ac_5_3F_ax_Neu5Ac is a metabolic inhibitor of sialyltransferases that blocks the addition of sialic acids to the glycan backbone (36).

**Sialic Acid Linkages**

**Figure 2:**
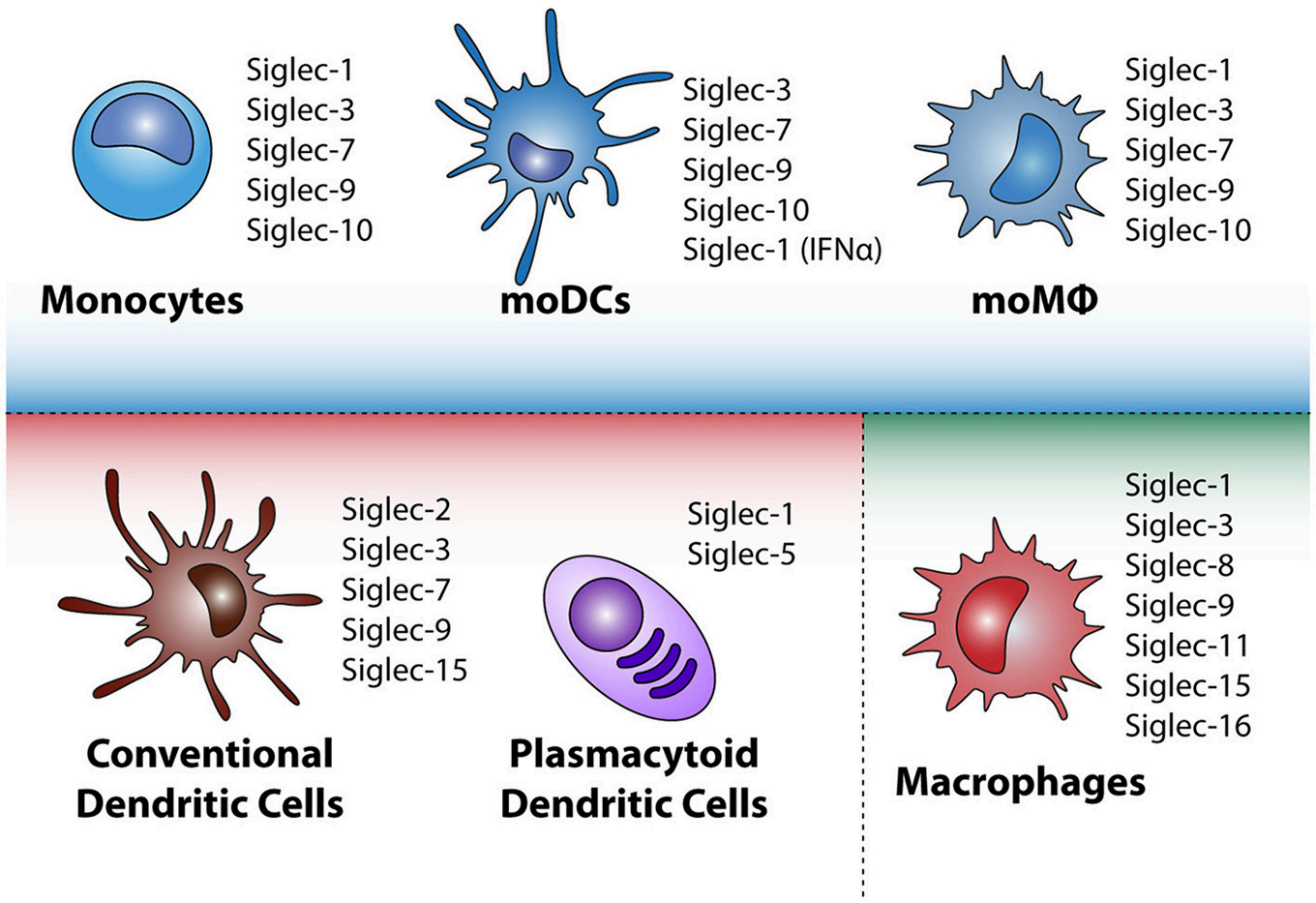
Siglec expression by different mononuclear phagocytes in steady state. Depicted in the blue square are monocytes and *in-vitro* cultured monocyte-derived dendritic cells (moDC) and monocyte-derived macrophages (moMQ). In the red square the conventional dendritic cells and plasmacytoid dendritic cells that appear in peripheral blood are highlighted, whereas in the green square tissue resident macrophages (MQ) are depicted. Depending on microenvironmental triggers the immunogenic or tolerogenic status of the mononuclear phagocyte can change, leading to altered Siglec expression.

Sialic acids can be linked to the underlying glycan via different types of linkages, which affects their recognition by glycan-binding receptors, such as Siglecs. These linkages mainly have an alpha configuration and are defined by which carbon in the acceptor glycan is connected to the anomeric carbon in the Neu5Ac (carbon 2). When sialic acid is transferred to a different glycan, the bond can involve the carbon 3 or 6 in the acceptor rising to α2,3 or a α2,6 linkages, respectively, (33, 35) (Figure 2B). In poly-sialic acid structures, one Neu5Ac is added to a strain of sialic acids in an α2,8 linkage. The different Sialic acid linkages are depicted in the complementary figure to this box.

**Trans/Cis Interaction**

Siglecs can interact with sialic acid on a different cell or protein/particulate (*trans* interaction) or with sialic acids present on the same cell that expresses the receptor (*cis* interaction), as depicted in the figure complementary with this box. An illustration of a *trans* interaction is α2,3 linked sialic acids expressed by lung epithelium under inflammatory conditions and Siglecs present on neutrophils (37, 38) (Figure 1C). An example of a *cis* interaction is α2,3 linked sialic acid present on the cell surface of moDCs, which bind to a Siglecs present on the same moDCs (39) (Figure 1D).

**Degradation**

Specific glycosidases, called neuraminidases or sialidases, can hydrolyse the sialic acid from oligosaccharides. Present mainly in intracellular vesicles, these enzymes can be secreted, thereby changing the profile of sialylated structures present on the cell membrane. Their expression is dysregulated in many different types of cancer.

**Sialic Acid Immune Modulation**

Sialic acids can modulate the immune system in diverse ways through Siglecs, influence on antibody mediated clearance of pathogens and through complement. Sialylation of the antibody immunoglobulin A (IgA) interferes with the cell surface attachment of influenza A and mediates anti-viral activity of IgA (40). Sialic acids can also bind to complement regulator factor H and by this negatively regulate the complement alternative pathway (41–43).

## Siglecs

The human genome contains 14 different Siglecs, which can be divided into two groups based on their genetic homology among mammalian species. The first group is present in all mammals and consists of Siglec-1 (Sialoadhesin), Siglec-2 (CD22), Siglec-4 (MAG), and Siglec-15 (48–50). The second group consists of the CD33-related Siglecs that have evolved rapidly and therefore their repertoire differs between species. The CD33 related Siglecs are Siglec-3 (CD33),−5,−6,−7,−8,−9,−10,−11,−14, and −16 (51). Monocytes, moMQ and moDCs have largely the same Siglec profile (Figure 2), namely high expression of Siglec-3,−7,−9, low Siglec-10 expression and upon stimulation with IFN-α, also Siglec-1 (52–60) is expressed. In contrast, MQ have primarily expression of Siglec-1,−3,−8,−9,−11,−15, and−16 depending on their differentiation status (49, 52, 61, 62). cDCs express Siglec-3,−7, and−9, similar to moDCs, but in addition also express low levels of Siglec-2 and Siglec-15 (49, 63–67). pDCs are different in their expression of Siglecs, as they express Siglec-1 and Siglec-5 (54, 68). The presence of the Siglecs on mononuclear phagocytes is based on their steady state situation, however, microenvironmental triggers that change the maturation status of the cell, may influence the loss or gain of the expression of Siglecs. Downregulation of Siglecs-7 and Siglec-9 expression on moDCs is observed after stimulating moDCs for 48 h with LPS, however, on moMQ Siglec expression is not changed upon LPS triggering (54). Clearly, further research on the regulation of Siglec expression during cellular maturation is needed. Siglecs are also present on other immune cells [nicely reviewed by MacAuley et al. (69)], such as B cells, basophils, neutrophils, and NK cells, with different expression patterns for every cell subset.

Sialic acids, the ligands for Siglecs receptors, are widely expressed as they are exposed on the outermost end of glycosylated structures of glycoproteins expressed on immune and other cells in the body, secreted glycoproteins in tissues and blood and on extracellular matrix in tissues (70, 71). It is the glycosylation machinery of the cells that determines the type of sialic acids to be added on the carbohydrate backbone to be expressed by the glycoprotein (Box 1, Figure 1). A Siglec-expressing immune cell can bind to sialic acids present on another cell or secreted glycoprotein and this is called a *trans* interaction (72) (Figure 1). Siglec receptors can also bind sialic acids exposed on the same cell, called a *cis* interaction. Moreover, Siglec receptors have different binding affinities for different linkage and modifications of sialic acids (see Box 1 for more information about sialic acid). Most Siglecs have a preference for a particular sialic acid linkage, being either α2,3, α2,6, or α2,8-linked sialic acid but Siglecs may also show redundant specificity toward more linkages (52, 58).

## Immune modulation through Siglec SIGNALING

**Figure 3:**
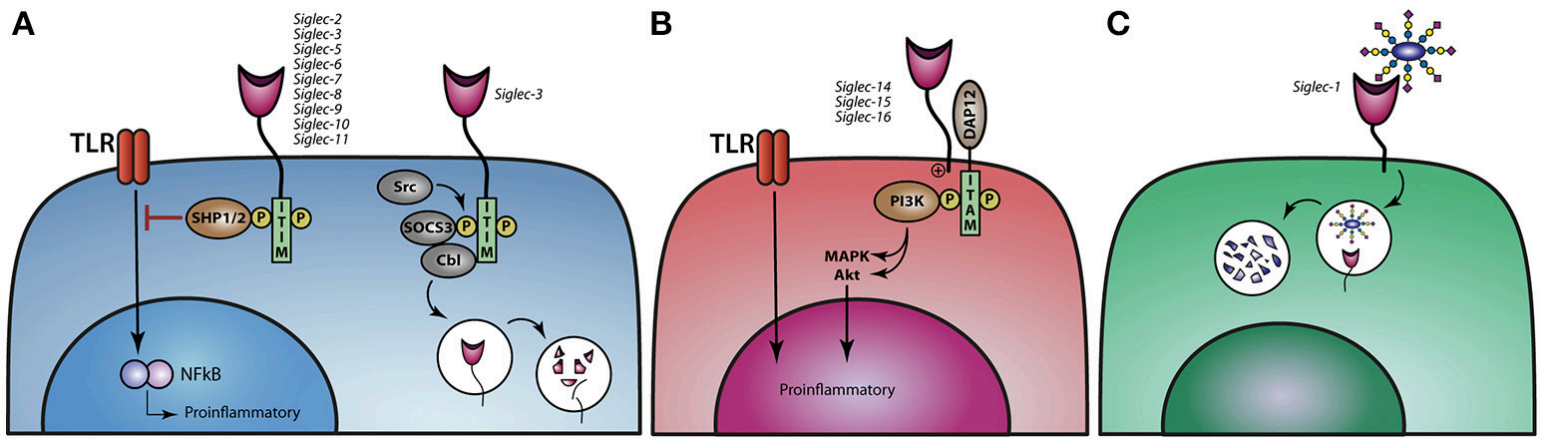
Siglec signaling and the immune modulatory effect on TLR signaling. **(A)** Siglec-2,−3, and−5 till−11 modulate TLR signaling upon binding of sialic acids and thereby dampen proinflammatory responses. Ligand binding to Siglec-3 leads to phosphorylation of the ITIM motif and reveals a binding site for SOCS3 and Cbl, causing proteasomal degradation of Siglec-3 and SOCS3. **(B)** Activating Siglecs-14,−15, and−16 can associate with DAP12, resulting in the activation of the MAPK and AKT pathways, thereby stimulating a proinflammatory response. **(C)** Siglec-1 can internalize upon binding of its ligand and thereby present antigens to dendritic cells or B cell to initiate an immune response.

The immune modulatory effect induced upon sialic acid binding to Siglec is regulated through downstream signaling pathways. Siglec-5 till Siglec-11, are the so-called inhibitory Siglecs, carrying ITIM and/or ITIM like motifs in their cytoplasmic domains, which can be phosphorylated by the Src family, thereby creating a binding site for the tyrosine phosphatases SHP-1 and SHP-2 (Figure 3A). Upon binding of SHP-1/2, de-phosphorylation of downstream targets can be achieved and ubiquitination, internalization, and phosphorylation of the receptor can be regulated (73, 74). The Src-mediated phosphorylation of ITIMs in Siglec-3 and possible also other ITIM-containing Siglecs can also lead to the binding of Cbl, a RING finger-containing E3 ligase, and suppressor of cytokine signaling 3 (SOCS3), resulting in the ubiquitination and protosomal degradation of Siglec-3. The same process also regulates the internalization and surface abundance of Siglec-3. SOCS proteins are upregulated by cytokines during inflammatory responses, leading to the loss of Siglec-3 and thereby higher proliferation of myeloid cells (75, 76) (Figure 3A). Signaling of different Siglecs through the binding of sialic acids or crosslinking via antibodies can lead to both an inflammatory or tolerogenic state in distinct mononuclear phagocytes. Antibodies against Siglec-3 and −7 inhibit the proliferation of myeloid cells (77) while monocytes treated with Siglec-3 antibodies show increased production of the pro-inflammatory cytokines IL-1β, TNF-α, and IL-8 (78). These findings illustrate that crosslinking of Siglec-3 expressed on different myeloid cells induces opposite functional outcomes. Moreover, crosslinking Siglec-3 on monocytes via antibodies signals a pro-inflammatory effect, while *cis* binding of sialic acids to Siglec-3 represses IL-1β production by monocytes (78).

In contrast, Siglec-4 and Siglec-14 till Siglec-16 do not have an ITIM or ITIM like motif, but instead signal through the association of DNAX activation protein (DAP)12 and are therefore called activating Siglecs. DAP12 associates with these activating Siglecs through a positively charged lysine residue in the transmembrane domains and contain a cytosolic immunoreceptor tyrosine-based activation motifs (ITAM), which can recruit PI3K (Figure 3B) (49, 62). Furthermore, Siglec-14 can promote an inflammatory response by activating the MAPK pathway (46). The activating Siglecs most likely developed under evolutionary pressure, when pathogens adapted using the inhibitory Siglecs to circumvent the immune system, allowing sialic acids to also activate the immune system. There are a couple of paired Siglecs, consisting of an inhibitory and an activating Siglec, like Siglec-5 and Siglec-14 as well as Siglec-11 and Siglec-16. Polymorphisms in Siglec-5/14 have been described, whereby the Siglec-14 gene is deleted and the Siglec-5 gene is present under the Siglec-14 promotor. When monocytes from these individuals are challenged with LPS or with group B *streptococcus* (GBS) they produce less TNF-α than individuals that have normal Siglec-5 and Siglec-14 expression on their monocytes, indicating that Siglec-14 is tipping the balance toward the pro-inflammatory site when cells are confronted by pathogens (46, 61, 62).

Siglec-1 is a non-signaling Sigelc, that internalizes upon ligand binding (Figure 3C). Den Haan et al. showed in mice that antigen coupled to Siglec-1 antibodies targets Siglec-1 expressing marginal zone macrophages that transfer antigen to CD8^+^ DC favoring effective antigen specific T cells to eradicate tumor growth (79, 80). Furthermore, it has been shown that Siglec-1^+^ MQ promote germinal center B cell responses upon Siglec-1 antibody targeting (81).

Siglecs can also exert their immune modulatory effects by altering TLR signaling. LPS stimulation of TLR4 induces CCR7 upregulation of moMQ which is inhibited by anti-Siglec-9 antibodies or knock down of expression of Siglec-9 (53, 82). G. Chen and colleagues revealed that TLR4 forms a complex with Siglec E (mouse homologue for human Siglec-7 and Siglec-9) in murine DCs and macrophages (83). This *cis* interaction between Siglec-E and TLR4 is likely mediated by sialic acids present on the TLR (83). The TLR-Siglec-E interaction, is abrogated by NEU1 (a lysosomal sialidase that cleaves sialic acids from their glycoprotein backbone), which is translocated to the cell membrane upon LPS stimulation.

moDCs treated with the Ac_5_3F_ax_Neu5Ac (Box 1), showed reduced sialic acid expression and a lower threshold of TLR activation, leading to increased sensitivity and response to poly I:C (TLR 3 agonist) and LPS, as reflected by the induction of moDC maturation and cytokine production by moDCs (55). Furthermore, it has been reported that sialic acid removal from moDCs uncovers Siglecs from their *cis* binding sialic acid ligands and increases expression of the maturation markers CD80 and CD86 and the secretion of IL-12 (84). Also, the cross presentation of melanoma antigens gp100 by DCs to antigen-specific CD8^+^ T cells was increased upon removal of sialic acid on moDC, illustrating that the presence of sialic acid constraints, that occupy Siglecs in *cis*, inhibits the effectiveness of moDC to induce immunity (84). Alternatively, targeting Siglecs with sialic acids or sialic acid mimetics in *trans* can modulate TLR signaling leading to a more tolerogenic DC phenotype. This illustrates that interference in the sialic acid-Siglec axis is central in the balance between immunity and tolerance.

## Sialic Acids Used by Pathogens to Modulate Immunity

The co-evolution of the immune system and pathogens has led to the acquisition of several strategies for pathogens to evade the immune system, which also includes the expression of sialylated glycans to induce tolerance. One of the most notable examples is *Trypanosoma cruzi*, a protist parasite responsible for Chagas disease. During its infective stage in vertebrates, called trypomastigote, *T. cruzi* expresses a unique enzyme called *trans*-sialidase that catalyses the reversible transference of sialic acid from host glycoconjugates to glycan structures on the surface of the parasite. By doing this, *T. cruzi* uses host glycans to mask its own antigens and to modulate anti-parasitic responses (85). Parasitic sialylated glycans can interact with Siglec-E [homolog of human Siglec 7 and 9, Siglec comparison between Mammalians was recently reviewed by Bornhöfft et al. (86)] in murine dendritic cells to suppress the production of the pro-inflammatory cytokine IL-12 (87). Moreover, the addition of sialic acid to the surface of the parasite results in a negatively charged coat that inhibits complement-mediated killing. Furthermore, thanks to the *trans*-sialidase activity, *T. cruzi* is also able to alter the sialylation status of CD8^+^ T cells, dampening their capacity to induce an effective anti-parasitic immune response (88).

Interestingly, several pathogenic bacteria also use the sialic acid-Siglec axis to dampen the immune system in favor of their survival. Despite the fact that sialic acids are mainly restricted to vertebrates, some bacteria have acquired the ability to take sialic acids or sialylated structures from the host, to synthetize “mimic” structures or even perform *de novo* synthesis of sialic acids, giving them a survival advantage. For example, Siglec-5 and−9 on neutrophils can be triggered by glycoconjugates present in *Pseudomonas aeruginosa* or *Group B streptococcus (GBS)* serotypes Ia and III, thereby inhibiting their ability to respond to the bacteria. Moreover, sialylated glycans present in *GBS* are able to inhibit the complement system, by reducing deposition of C3b on their surface and, therefore, the generation of C5a and the membrane attack complex (89–91).

The presence of sialic acids in envelope glycoproteins of viruses also contributes to enhanced infection of the host. This is the case for the *Human immunodeficiency virus* (HIV) and the *Porcine reproductive and respiratory syndrome virus* (PRRSV), which can bind to Siglec-1 to promote *trans* infection (92–94). Nevertheless, Siglec-1 ligands on *GBS* surface interact with Siglec-1 on marginal zone macrophages for the subsequent generation of anti-*GBS* immune responses (95).

Influenza A virus recognizes α2,3 and α2,6 linked sialic acids with its hemagglutinin (HA) glycoproteins to infect host cells. On the other hand, influenza A virus carries the neuraminidase (NA) glycoprotein that can cleave off sialic acids from cellular and viral glycoproteins that are expressed in infected cells and assembled in virions, to reduce HA causing aggregation of the virions to the cell surface. The HA and NA proteins are in perfect balance to warrant infection and to abolish detection by the immune system (96). Another example is the non-typeable *Haemophilus influenzae* (NTHi), which is also able to take up sialic acids through a tripartite ATP-independent periplasmic (TRAP) transporter. Incorporation of the sialic acids in the NTHi membrane protects it from serum-mediated killing (97).

Sialic acids are used by different pathogens to infect host cells and dampen the immune response. Knowledge on this mechanism can be exploited to design new therapeutic strategies in cancer or auto-immune diseases and asthma.

## Sialic Acid—Siglec Axis in Cancer

**Figure 4:**
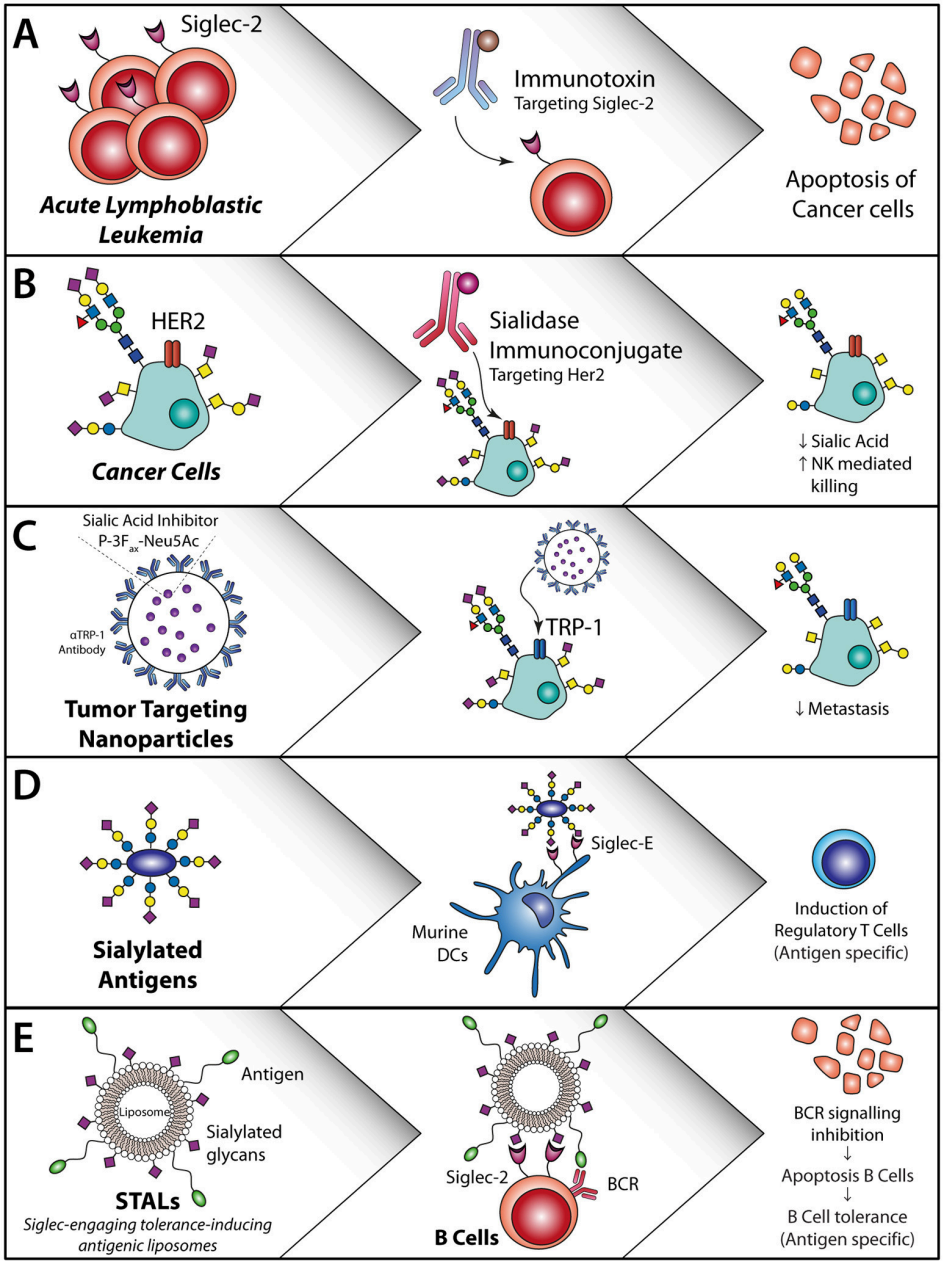
Sialic acid—Siglec axis usage for the treatment of cancer or allergies and auto-immune diseases. **(A)** Siglec-2 antibodies coupled to an immunotoxin induce apoptosis of Siglec-2-expressing acute lymphoblastic leukemia cells (104). **(B)** HER2 targeting with a sialidase coupled to the HER2 antibody or locally applied non-targeted sialidases/synthesis inhibitors. This decreases sialic acid expression, reduce T reg induction and induced T cell activation and initiates NK cell killing (105). **(C)** Sialic acid inhibitor P-3F_ax_-Neu5Ac inclusion in nanoparticles targeted to tumor cells inhibits the sialic acid expression on the tumor cells, thereby decreasing metastasis and increasing tumor cell killing (106, 107). **(D)** Sialylated antigens target DC to remove regulatory T cells (108). **(E)** Antigen-specific B cell apoptosis induction by STALs targeting Siglec-2 in combination with an antigen that inhibits B cell receptor signaling on B cells (109).

Aberrant glycosylation of multiple cancers and its influence on cancer progression and metastasis are well-known. Increased sialylation, α2,3; α2,6, and α2,8 linked sialic acids, has been demonstrated in multiple tumor tissues like renal cell carcinoma, prostate cancer, colon cancer, breast cancer, head and neck squamous cell carcinoma and oral cancer (98–101). This aberrant sialylation can also be detected in serum serving as potential biomarkers for cancer detection, progression and treatment responses (99, 101–103) (Figure 4).

In a mouse model for melanoma, hyper sialylation of B16 melanoma cells leads to increased tumor growth, associated with an enhanced T regulatory/T effector balance and reduced NK cell activity within the tumor and secondary lymphoid organs (110). DCs that interacted and sampled sialylated antigens via Siglec-E (murine homologue of human Siglec-7 and Siglec-9) induced regulatory T cells and inhibited effector T cell function *in-vivo*. These findings revealed that tumor sialylation impedes T cell-mediated anti-tumor immune responses, while promoting tumor-associated regulatory T cells (110). Blocking the inhibitory effects of sialic acids with a sialic acid blocking glycomimetic (Box 1) in a B16-OVA mouse model revealed reduced tumor growth, enhanced tumor killing by ovalbumin specific CD8^+^ T cells and inhibition of metastasis (106, 107) (Figure 4C).

In breast cancer a specific glycoform of transmembrane mucin 1, MUC1-T is sialylated, creating MUC1-sT (111, 112). The MUC1-sT can interact with Siglec-9 on monocytes and thereby induce secretion of IL-6, M-CSF and chemokines associated with tumor progression. Binding of MUC1-sT to Siglec-9 on macrophages induces a tumor-associated macrophage (TAM) phenotype, that inhibits CD8^+^ T cell proliferation and results in the upregulation of IDO, CD163 and PD-L1 *in-vivo* (113, 114). Another specific mucin glycoform, called MUC2-sT, has been shown to increase apoptosis of immature moDCs (115). Together, this points toward a broad immunological suppression by tumor-produced sialylated mucins.

Antibodies against Siglecs are explored for the treatment of different cancer types. For Acute Lymphoblastic Lymphoma (ALL) the FDA approved Inotuzumab Ozogamicin (Besponsa®), a monoclonal antibody against Siglec-2 coupled to the toxic agent calicheamicin is used. This antibody targets Siglec-2 positive B-lymphoblasts and causes cell death of these cells through the toxic agent (Figure 4A). Trials with this antibody revealed that an enhanced number of patients reached complete remission and had an increased overall progression free survival. However, serious adverse effects were seen like myeloid suppression (104), which could be due to the presence of Siglec-2 on DC subsets. Another Siglec that is targeted for the treatment of acute myeloid leukemia (AML), is Siglec-3, using Siglec-3 antibody coupled to calicheamicin (116, 117). Hereby, the myeloid blasts that express Siglec-3 are targeted and this improved outcome in patients with relapsing disease as well as in elderly patients that were not eligible for extensive chemotherapy (116). Similar to the Siglec-2 antibody treatments, the Siglec-3 antibodies caused extensive adverse effects, probably due to the wide spread expression of Siglec-3 on (healthy) myeloid subsets. Therefore, it is of great imporatnce to have a complete and accurate overview of Siglec expression on immune cells. Other strategies to target Siglec-3 in AML include the use of CAR-T cells. Siglec-3 targeting CAR T-cells have shown to induce CD8^+^ T cell degranulation against primary AML and AML cell lines *in-vitro* (118, 119). Although different CAR-T cells are already tested in the clinic for lymphoid leukemia (120), it is questionable whether Siglec3 targeting CAR-T cells have similar severe side effects as observed with Siglec-3 antibodies.

Instead of targeting the Siglecs using antibodies, modifying the phosphorylation status of Siglec-3 and Siglec-9, in particular dephosphorylation of the receptos, has shown to lead to increased immunity of moDCs, when treated with Dasatinib a SRC tryrosine kinase inhibitor that dephosphorylates Siglec-3 and Siglec-9 (121). Also, leukemia (BCR-ABL^+^ AML) patients treated with Dasatinib, had a stronger CD8^+^ T cell and NK cell response associated with long lasting remission (122). Another strategy to increase anti-tumor immunity through Siglecs has been developed by Xiao et al., where they target HER2 with a monoclonal antibody fused to a sialidase (105) (Figure 4B). This sialidase specifically cuts off the sialic acid ligands that are bound by Siglec-7 and Siglec-9 and thereby increase NK activity. *In vitro* these HER2 targeting antibodies fused to a sialidase, increased the NK cell mediated killing of HER2 positive tumor cells (105). As most breast cancer patients are HER2 positive, targeting of HER2 with this sialidase fused antibody could be an effective treatment strategy.

Most strategies that interfere with the sialic acids-Siglec Axis are developed for leukemic cells as they have high expression of Siglec-2 or Siglec-3 and are therefore easily targetable. Other cancer type treatments could also benefit from targeting Siglecs, blocking of Siglecs could abrogate the inhibitory effects on mononuclear phagocytes and lead to better migration and maturation of these cells, which subsequently stimulates tumor-specific T cell responses. Moreover, local removal of tumor-associated sialic acid may temporarily de-tolarize the tumor microenvironment and trigger immune activation at the tumor site. A combination with checkpoint inhibitors would than favor improved tumor eradication.

## Sialic Acid—Siglec Axis to Induce Tolerance for Allergies and Auto-Immune Diseases

While in cancer it is important to induce immunity, in allergies and auto-immune diseases, an overactive immune system needs to be restored by inducing tolerance. Exploring the potential of the Sialic acid-Siglec Axis is an alternative to induce tolerance in an antigen specific manner. Because immune inhibitory Siglecs are found on mononuclear phagocytes, strategies can be designed aimed to actively induce tolerance via targeting inhibitory Siglecs on mononuclear phagocytes.

Modification of antigens such as OVA or MOG peptides with α2,3 or α2,6 sialyl-lactose has shown to increase targeting of these antigens to Siglec E, the human Siglec 7 and Siglec-9 homologue, and alter DC function in mice. Both *in-vitro* and *in-vivo* experiments demonstrated that sialic acid modified antigens induced antigen specific T reg induction and inhibition of inflammatory effector cells when activated with LPS (108) (Figure 4D).

Also, Siglec-engaging tolerance-inducing antigenic liposomes (STALs) are employed, in which sialic acid decorated nanoparticles, or sialo-glycoproteins or Siglec antibody targeting are used for Siglec targeting to induce tolerance. STALs with the peanut allergen Ara h2 (Ah2) and a high affinity Siglec-2 ligand (modified α2,6 linked sialic acid) incorporated in the outer membrane have shown high binding affinity to the B cell receptor and Siglec-2 simultaneously and to prevent peanut allergy against the Ah2 allergen in mice (109) (Figure 4E). This is acclaimed to the forced interaction between the B cell receptor and Siglec-2, thereby inducing apoptosis of autoreactive B cells. Pang et al. used the same STALs and incorporated rapamycin in the STALs and thereby enhanced the tolerogenic capacity in mice, which was the result of increased phagocytosis of these STALs by macrophages and DCs (123).

Sialic acid mimetics, such as the modified sialic acid coupled to liposomes discussed above, comprise of natural sialic acids as a backbone and are modified at certain positions in the sialic acid structures to develop high affinity ligands for Siglecs (124–126). Addition of hydrophobic groups at the C2 and C9 of α2,6 sialic acids results in a high affinity Siglec-2 ligands, which out-competing the *cis* interaction between the Siglec and its ligand. As a result better binding, endoyctosis, and eventually apoptosis of targeted B cells is acquired (126, 127).

Nanoparticles decorated with α2,8 linked sialic acids were developed to target murine Siglec-E (homologue of human Siglec-7 and Siglec-9) on macrophages. This approach limited the pro-inflammatory cytokines production by LPS-treated MQ *in-vitro*. Subsequently, these nanoparticles were able to limit the inflammation and increase levels of IL-10 in serum in a mouse model for LPS-induced systemic inflammation. Similar results were seen with human moMQ, resulting in an anti-inflammatory cytokine profile. In an *ex-vivo* human lung perfusion model the nanoparticles coated with α2,8 linked sialic acids reduced pulmonary oedema after LPS-induced injury (128).

Several studies have shown the importance of Siglec-sialic axes in auto-immune disease and allergy due to expression of Siglecs on other immune cells such as eosinophils and B-cells. Asthma is an eosinophil, expressing Siglec-8, mediated disease and it has been shown that polymorphisms in the SIGLEC8 gene are linked to the development of asthma (38, 129). Antibodies against Siglec-8 or the mouse homolog Siglec-F induce caspase and ROS dependent apoptosis of eosinophils (130, 131). Autoantibodies against Siglec-8 have been found in intravenous immunoglobulin preparations that are used in various chronic inflammatory disorders, although some cytotoxic effects are known (132). For asthma it would be beneficial to have specific Siglec-8 agonists to induce neutrophil apoptosis without the risk of side effects observed with intravenous immunoglobulin injections. Another example is the anti-Siglec-2 antibody (Epratuzumab) that has already been tested in seven clinical trials for the autoimmune disease systemic Lupus erythematosus (SLE). Although initial trials showed promising effects with reduced peripheral B cell numbers, the overall effect was not better than standard care for SLE (reviewed by D Geh) (133).

Most of these strategies are to date only tested in *in-vitro* or *ex-vivo* experiments and should be tested in *in-vivo* and clinical trials as they have great potential for future applications in the treatment of allergies and auto-immune diseases. It is also important to elucidate the Siglec expression on different human immune cell subsets in order to identify the potential risks on side-effects by targeting multiple Siglecs with one ligand.

## Concluding Remarks

The last decade researchers identified the enormous potential of the sialic acid—Siglec axis to induce wanted or unwanted immune tolerance in cancer, allergies or auto-immune diseases. Both Siglec targeting antibodies, sialic acid mimetics, or glycan modifying agents can be used to interfere in this process and open new area's in the design of novel therapies for cancer, allergy and auto-immune diseases.

Still several questions need to be answered related to a better understanding of the biology of the Siglec-sialic acid axis. Those relate to the signaling capacity that sialic acid impose on immune cells to modify its function toward tolerance induction or activation of immunity. Interesting research topics to be addressed are: How do the various Siglecs expressed on one cell communicate with each other What is the exact specificity of these receptors for sialic-acids? Does multivalency of sialic acids or Siglec receptors matter? Other intriguing questions to be solved are: Do we need to inhibit only one Siglec receptor or more Siglec receptors simultaneously on one cell to alter function? What is the relation to *cis* and *trans* interaction on Siglec-sialic acid interactions? How important is the protein or lipid backbone on which the sialic acid is exposed? To answer these questions an urgent need for Siglec specific targeting molecules is needed, which can be sialic acid mimetics or Siglec specific antibodies.

As the Siglec—sialic axis plays a crucial role in tissue homeostasis and the resolution of inflammation, more studies are necessary to understand their involvement in these biomedical processes. A better understanding of its role in the resolution of inflammation is crucial for its application in the treatment of auto-immune diseases and allergies.

Moreover, both pathogens and tumors use the Siglec-sialic acid axis in their own benefit. It is therefore of vital importance to design new methods to modify glycosylation at site of infection or tumor location. Several studies already touch upon the investigation of targeting specific sialidases to the tumor micro-environment to remove the sialic acid content involved in the induction of tolerance in the tumor microenvironment. To unleash the sialic acid imposed tolerance in the local tumor microenvironment may be combined with other immune checkpoint inhibitors to stimulate tumor immunity in a multilevel manner. Alternatively, presence of sialic acid signatures in the tumor microenvironment may serve as new biomarkers to define immune tolerizing signatures in individual tumors and response therapy prediction (99).

Future studies are of great importance to unveil the complex Siglec-sialic acid axis and will warrant new discoveries in clinical application strategies in cancer, allergy and auto-immune diseases.

## Author Contributions

JL was involved in the writing, reading of literature, design and discussion of the figures. ER was involved in writing, critical reading of the manuscript and designing the figures. YvK was involved in the overall supervision of the review and editing of the manuscript.

### Conflict of interest statement

The authors declare that the research was conducted in the absence of any commercial or financial relationships that could be construed as a potential conflict of interest.
